# c-Myc Drives inflammation of the maternal-fetal interface, and neonatal lung remodeling induced by intra-amniotic inflammation

**DOI:** 10.3389/fcell.2023.1245747

**Published:** 2024-02-28

**Authors:** April W. Tan, Xiaoying Tong, Silvia Alvarez-Cubela, Pingping Chen, Aline Guimarães Santana, Alejo A. Morales, Runxia Tian, Rae Infante, Vanessa Nunes de Paiva, Shathiyah Kulandavelu, Merline Benny, Juan Dominguez-Bendala, Shu Wu, Karen C. Young, Claudia O. Rodrigues, Augusto F. Schmidt

**Affiliations:** ^1^ Division of Neonatology, Department of Pediatrics, University of Miami Miller School of Medicine/Holtz Children’s Hospital, Miami, FL, United States; ^2^ Diabetes Research Institute, University of Miami Miller School of Medicine, Miami, FL, United States; ^3^ Department of Biomedical Science, Florida Atlantic University Charles E. Schmidt College of Medicine, Boca Raton, FL, United States; ^4^ Department of Molecular and Cellular Pharmacology, University of Miami Leonard M. Miller School of Medicine, Miami, FL, United States; ^5^ Division of Pediatric Nephrology, Department of Pediatrics, University of Miami Miller School of Medicine, Miami, FL, United States; ^6^ Interdisciplinary Stem Cell Institute, University of Miami Leonard M. Miller School of Medicine, Miami, FL, United States

**Keywords:** intra-amniotic inflammation, preterm birth, bronchopulmonary dysplasia, pulmonary hypertension, placental inflammation, fetal inflammation

## Abstract

**Background:** Intra-amniotic inflammation (IAI) is associated with increased risk of preterm birth and bronchopulmonary dysplasia (BPD), but the mechanisms by which IAI leads to preterm birth and BPD are poorly understood, and there are no effective therapies for preterm birth and BPD. The transcription factor c-Myc regulates various biological processes like cell growth, apoptosis, and inflammation. We hypothesized that c-Myc modulates inflammation at the maternal-fetal interface, and neonatal lung remodeling. The objectives of our study were 1) to determine the kinetics of c-Myc in the placenta, fetal membranes and neonatal lungs exposed to IAI, and 2) to determine the role of c-Myc in modulating inflammation at the maternal-fetal interface, and neonatal lung remodeling induced by IAI.

**Methods:** Pregnant Sprague-Dawley rats were randomized into three groups: 1) Intra-amniotic saline injections only (control), 2) Intra-amniotic lipopolysaccharide (LPS) injections only, and 3) Intra-amniotic LPS injections with c-Myc inhibitor 10058-F4. c-Myc expression, markers of inflammation, angiogenesis, immunohistochemistry, and transcriptomic analyses were performed on placenta and fetal membranes, and neonatal lungs to determine kinetics of c-Myc expression in response to IAI, and effects of prenatal systemic c-Myc inhibition on lung remodeling at postnatal day 14.

**Results:** c-Myc was upregulated in the placenta, fetal membranes, and neonatal lungs exposed to IAI. IAI caused neutrophil infiltration and neutrophil extracellular trap (NET) formation in the placenta and fetal membranes, and neonatal lung remodeling with pulmonary hypertension consistent with a BPD phenotype. Prenatal inhibition of c-Myc with 10058-F4 in IAI decreased neutrophil infiltration and NET formation, and improved neonatal lung remodeling induced by LPS, with improved alveolarization, increased angiogenesis, and decreased pulmonary vascular remodeling.

**Discussion:** In a rat model of IAI, c-Myc regulates neutrophil recruitment and NET formation in the placenta and fetal membranes. c-Myc also participates in neonatal lung remodeling induced by IAI. Further studies are needed to investigate c-Myc as a potential therapeutic target for IAI and IAI-associated BPD.

## 1 Introduction

Approximately 15 million infants are born preterm annually worldwide, and preterm birth is the leading cause of death in children under the age of 5 years (Blencowe et al., 2012; Liu et al., 2016). Survivors of preterm birth suffer a lifetime of disability and continue to require complex multidisciplinary medical care beyond the neonatal period and childhood years. Adult survivors of preterm birth are at increased risks for mortality, chronic multi-organ diseases, psychiatric disorders, and decreased quality of life (Crump et al., 2019; Markopoulou et al., 2019; Crump, 2020; Petrou et al., 2020). Intra-amniotic inflammation (IAI) is present in 70%–85% of preterm births that occur before 30 weeks of gestation and is the cause of up to 40% of preterm births (Romero et al., 1998; Romero et al., 2014). Current clinical strategies to manage IAI include the use of antibiotics and expectant management of labor, but IAI is most often sterile, and the poor clinical outcomes of preterm birth and neonatal morbidities are associated with the presence of inflammation itself, regardless of bacterial infection (Combs et al., 2014).

The presence of IAI is also associated with increased risk for bronchopulmonary dysplasia (BPD) in preterm infants, compounding the severe chronic morbidities that survivors of preterm birth already face (Villamor-Martinez et al., 2019). Moreover, the risk of BPD is inversely proportional to gestational age. With advances in neonatology over the past decade, more extremely low gestational age and extremely low birth weight infants are surviving, but the prevalence and burden of long-term impairment from prematurity and BPD have also increased. There is a lack of effective therapies for BPD, and BPD continues to be the most common long-term morbidity among preterm infants leading to lifelong respiratory impairment (Stoll et al., 2015). Survivors of preterm birth with BPD experience more childhood wheezing and respiratory illnesses and have more special care needs (Stoll et al., 2015; DeMauro, 2018). Adult survivors of BPD have altered lung structure, impaired lung function and exercise capacity, and decreased quality of life (Caskey et al., 2016). Preterm birth and BPD are major public health issues, hence there is a pressing need for effective targeted therapies to prevent preterm birth and BPD.

The pathogenesis of BPD is multifactorial and involves multiple pathways, posing a challenge to the development of new therapies (Mathew, 2020). c-Myc is an oncogene and key transcription factor that regulates multiple cellular functions including proliferation, differentiation, cell metabolism and apoptosis. c-Myc is downstream of multiple pathways that have been implicated in the pathophysiology of both preterm birth and BPD such as tumor necrosis factor-α (TNFα), Notch signaling, Wingless/Int-1 (Wnt) signaling, and Janus Kinase/Signal transducers and activators of transcription (JAK/STAT) signaling (Green and Arck, 2020; Mathew, 2020). c-Myc has a basic-helix-loop-helix-leucine zipper structure and heterodimerizes with a ubiquitous protein called Max to become transcriptionally active (Chen et al., 2018). In tracheal aspirates of preterm infants, the MYC/MAX complex was overrepresented in lung macrophages of infants prone to BPD (Sahoo et al., 2020). We hypothesize that c-Myc has a role in modulating inflammation of the placenta and fetal membranes in IAI, leading to fetal lung inflammation and neonatal lung remodeling. To test our hypothesis, our first objective was to determine the kinetics of c-Myc in the placenta, fetal membranes and lungs exposed to IAI in a pregnant rat model of IAI using ultrasound-guided intra-amniotic lipopolysaccharide (IA LPS) injections. We then prenatally treated pregnant rats with IAI induced by IA LPS with a small molecule c-Myc inhibitor 10058-F4 (Huang et al., 2006). We show that c-Myc inhibition in a rat model of IAI decreased inflammation in the placenta and fetal membranes, and attenuated lung parenchymal and vascular remodeling induced by IAI, demonstrating a potential role of c-Myc in modulating inflammation at the maternal-fetal interface, and neonatal lung remodeling induced by IAI.

## 2 Materials and methods

### 2.1 Animal model of IAI

**FIGURE 1:**
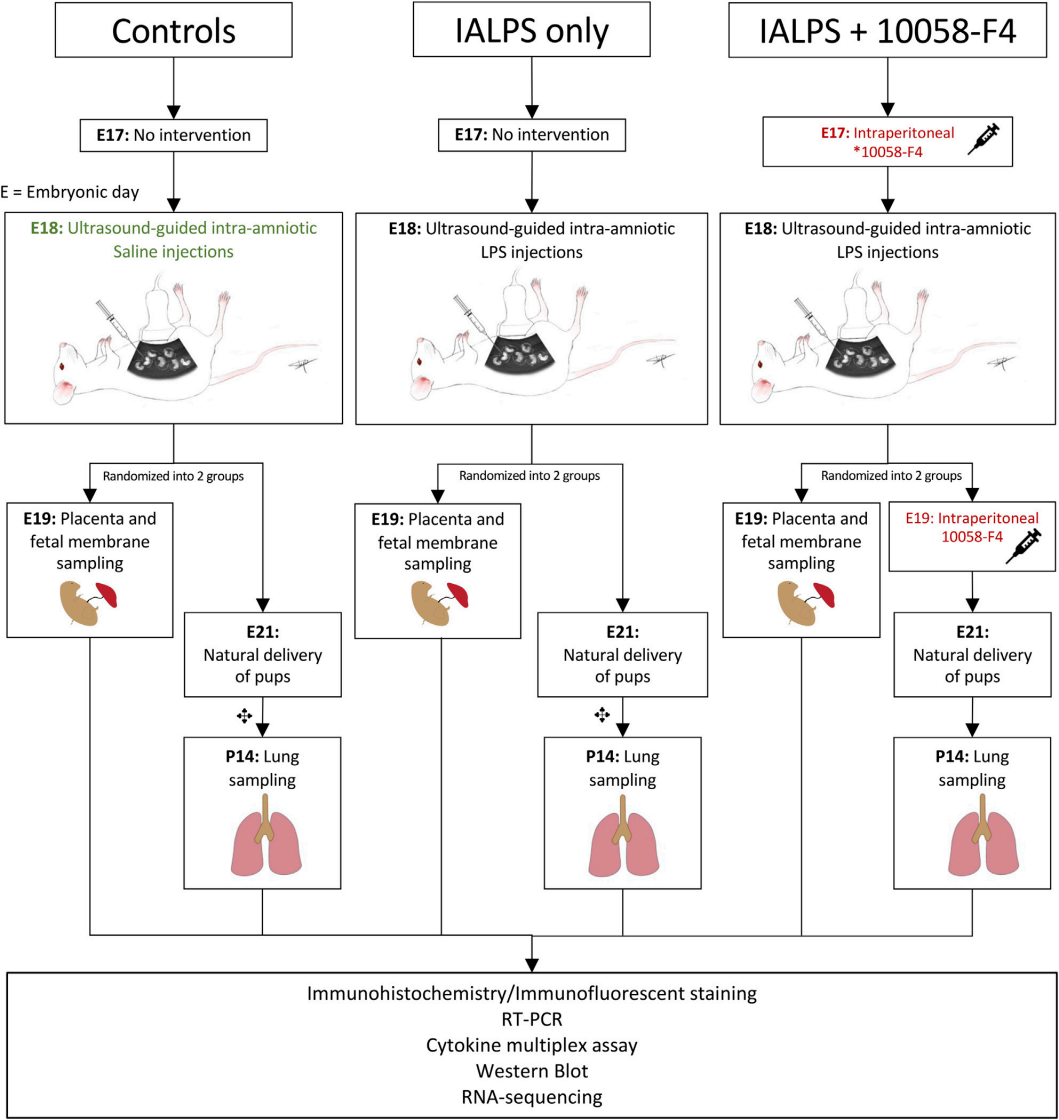
Experimental design. Pregnant time-mated Sprague-Dawley rats were randomized into 3 groups: 1) IA saline, 2) IALPS, or 3) IALPS +10058-F4. Intra-amniotic injections were performed on E18 under ultrasound guidance. Intraperitoneal c-Myc inhibitor injections were administered on E17 only for pregnant rats who underwent C-section for placenta and fetal membrane sampling on E19, and both E17 and E19 for pregnant rats undergoing natural delivery of pups for neonatal lung sampling. ✥subgroup of rat pups euthanized at P0, P3 and P7 for assessment of c-Myc expression over postnatal lung development. IALPS = Intra-amniotic lipopolysaccharide, E = Embryonic day. *10058-F4 = c-Myc inhibitor.

To determine the kinetics of c-Myc in normal lung development and in IAI-exposed animals, time-mated Pregnant Sprague-Dawley rats received ultrasound-guided (Vevo 3100, VisualSonics) intra-amniotic injections of 10 μg lipopolysaccharide (*E. coli* O55:B5, cat. #L4525-5MG, Sigma-Aldrich, St Louis, MO) (IA LPS) or sterile phosphate buffered saline (PBS) at embryonic day 18 (Figure 1). A group of animals were delivered by cesarean sections 3 h after IA LPS injections for *in vivo* imaging of LPS distribution using a Cy5.5-tagged LPS (cat # LPS-S55-1, Nanocs, Boston, MA) and imaged on IVIS Spectrum *In Vivo* Imaging System (PerkinElmer Inc., Waltham, MA). For assessment of IAI, a group of animals were delivered by cesarean section at 24 h post-IA LPS for placenta and fetal membrane sampling. Another group of animals were allowed to deliver naturally around embryonic day (E) 21 and rat pup lungs were sampled at postnatal day 14. For assessment of c-Myc lung expression in postnatal development, a subgroup of animals was sacrificed at four timepoints: day of delivery (P0), and postnatal days (P) 3, 7 and 14.

### 2.2 Treatment groups

For c-Myc inhibition experiments, time-mated pregnant Sprague-Dawley rats were randomized into three groups: IA saline injections (control); IA LPS injections only, or IA LPS injections with c-Myc inhibitor (IA LPS+10058-F4) (Figure 1). 10058-F4 (MedChemExpress, NJ, United States) was diluted per manufacturer’s instructions in 10% dimethyl sulfoxide (DMSO) and 90% sesame oil to 10 mM. One group of pregnant dams received intraperitoneal injections of 20 mg/kg of 10058-F4 on E17. Placenta and fetal membranes were sampled and analyzed at 24 h post-IALPS injections after 1 dose of 10058-F4. The remaining group of pregnant rats received a second dose of 10058-F4 on E19 and were allowed to naturally deliver. Rat pups were euthanized and neonatal lungs analyzed on P14.

### 2.3 Histological assessment

Whole placentas with fetal membranes and whole fetuses were fixed with 4% paraformaldehyde overnight. Following fixation, samples were embedded in paraffin and sectioned. Hematoxylin/eosin (H&E) staining was performed to assess for neutrophil infiltration in whole sections of placenta and fetal membranes. TUNEL assay was used to assess apoptosis in placenta and fetal membranes using a commercial kit (Click-iT Plus TUNEL Assay, cat #10617, ThermoFisher, Waltham, MA) according to the manufacturer’s instructions. To assess differences in cell proliferation, placenta sections were stained with Ki67 antibody and total number of cells and number of Ki67-stained cells per high power field (hpf) were quantified using Zeiss Axio Observer Microscopy image processing software. Six distinct regions of two placenta sections per sample was analyzed. Analysis was performed by calculating ratio of cells stained with Ki67 to total number of cells per hpf.

Neonatal lungs were inflation-fixed with 4% paraformaldehyde at 30 cm H_2_O via a tracheal cannula for 5 min and then fixed overnight. Following fixation, samples were embedded in paraffin and sectioned. To assess lung alveolarization in the peripheral parenchymal regions of lungs, lung sections were stained with H&E, and mean linear intercept (MLI) was performed on six distinct regions of one lung section per animal, avoiding large vessels and airways, and was calculated as previously described (Knudsen et al., 2010). Radial alveolar counts were performed on ten regions of one lung section per animal and calculated as previously described (Cooney and Thurlbeck, 1982). Pulmonary vascular muscularization was assessed by calculating ratio of small pulmonary vessels identified by endothelial cells staining with vWF that simultaneously stained positive for smooth muscle actin (SMA) antibody, as previously described (Ciuclan et al., 2011). Peripheral parenchymal regions of lungs were analyzed to avoid large vessels and airways. 4–5 animals were assessed per group. We performed immunohistochemistry on paraffin-embedded tissue sections with heat-assisted antigen retrieval with citrate buffer (pH 6.0). Primary antibodies (Supplementary Table S1A) were incubated overnight at 4°C followed by incubation with respective secondary antibodies for 1 h at room temperature. Stained sections were imaged on Zeiss AxioObserver microscope.

### 2.4 Cytokine/chemokine assay

Rat cytokine/chemokine concentrations in whole lung protein extract from 5 animals per group was determined by rat cytokine array/chemokines array-27 (Eve Technologies, Calgary, Canada). Flash frozen whole lung tissues were homogenized in RIPA buffer (Santa Cruz Biotechnology, catalog # sc-24948) and centrifuged at 12,000 rpm for 20 min at 4°C. The supernatant was transferred to a new tube and protein concentration was measured by BCA protein assay (Thermo Scientific, catalog # 23228 and 1859078). Samples were then diluted for a target protein concentration of 3–4 mg/mL. Values for samples with signal outside the curve were calculated when feasible by the model.

### 2.5 Western blot analyses

Whole placentas with fetal membranes were sectioned into equal quarters and homogenized in RIPA lysis buffer. Homogenates were centrifuged at 18,000 × g for 5 min at 4°C and the supernatant collected for protein analysis. An aliquot of each sample was used for protein quantification by the Bradford method, using a commercial kit (Bio-Rad Protein Assay Dye Reagent, Bio-Rad Laboratories Inc., United States). For Western blot analysis, 40 μg of total protein from each sample were fractionated by SDS-PAGE on precast 4%–15% Tris-glycine gradient gels, and then transferred to a 0.45 μm nitrocellulose membrane (Bio-Rad Laboratories, Inc., United States). Total protein was stained using the Revert 700 Total Protein stain kit (LI-COR Biosciences, United States) followed by imaging. Subsequently, the membrane was blocked overnight at 4°C under gentle agitation in Phosphate Buffered Saline pH 7.4 with 0.1% Tween-20 (PBS-T) supplemented with 5% nonfat dry milk (Bio-Rad Laboratories Inc., United States). After blocking, the membrane was washed in PBS-T and incubated for 1 h with mouse monoclonal anti-c-Myc antibody (clone 9E10, Santa Cruz Biotechnology, Inc. United States) diluted 1:500 at room temperature under gentle agitation. The membrane was then washed and incubated with IRDye^®^ 800CW Goat anti-Mouse IgG Secondary Antibody (LI-COR Biosciences, United States). All images were collected using the Odyssey^®^ Infrared Imaging System (LI-COR Biosciences, United States). Protein expression was estimated relative to total protein using Empiria Studio 2.3 software (LI-COR Biosciences, United States). Lung lysates were processed and analyzed in the same manner, with the exception that secondary antibody conjugated to horseradish peroxidase was used and proteins detected by chemiluminescence (Amersham, Piscataway, NJ, United States). Protein expression was estimated by densitometry analysis relative to actin expression using Quantity One software (Bio-Rad Laboratories Inc., United States). We analyzed 6–8 animals per group for placenta and fetal membrane analysis, and 4–7 rat pups per group for lung analysis.

### 2.6 RNA isolation and real-time qPCR

Total RNA was extracted from frozen placenta and lung tissues using the RNeasy universal Mini Kit (Qiagen, Valencia, CA) according to the manufacturer’s instructions. Two µg of total RNA from 4 to 7 animals per group was reverse-transcribed in a 20 µL reaction by using High-Capacity RNA-to-cDNA™ Kit according to supplier’s protocol (Applied Biosystems, cat #43-874-06, Foster City, CA) The real-time q-PCR was performed on an ABI Fast 7500 System (Applied Biosystems, Foster City, CA). Each reaction included diluted first-strand cDNA, target gene primers, or 18S rRNA gene primers and master mix containing TaqMan probes according to the supplier’s instruction (Supplementary Table S1B) (Applied Biosystems, Waltham, MA). The expression levels of target genes were normalized to 18S rRNA.

### 2.7 RNA sequencing

RNA quality and integrity were verified using the Agilent 2,100 Bioanalyzer (Agilent Technologies). All samples had RNA integrity number >8. RNA-Seq was performed by BGI genomics with a read depth of 30 million reads per sample for 150 bp paired-end reads. The raw sequence reads in FASTQ format were aligned to the *Rattus norvegicus* genome build rno6.0 using kallisto (Bray et al., 2016) followed by gene summarization with tximport (Soneson et al., 2015). After checking data quality, differential expression analyses comparing treatment groups with control and between each other were performed using DESeq2 with false discovery adjustment (Love et al., 2014). Genes were considered differentially expressed based on their fold change relative to control (≥1.5), *p*-value (<0.05), and q value (<0.1). PCA analysis was performed with PCAtools (Blighe and Lun, 2023). Volcano plots were generated using the EnhancedVolcano package (Blighe et al., 2018) Heatmaps were generated with pheatmap (Kolde, 2019).

### 2.8 Functional enrichment and pathway analysis

Lists of differentially expressed genes were used for functional enrichment analysis of Gene Ontology (GO) and KEGG pathway terms using the ToppCluster web server (Kaimal et al., 2010). Only unique terms associated with either induced or suppressed genes and at least 2 genes are reported. Negative log *p* values represent terms associated with suppressed genes, and positive log *p* values are associated with induced genes.

## 3 Results

### 3.1 US-guided IA LPS injections accurately target the amniotic cavity

**FIGURE 2:**
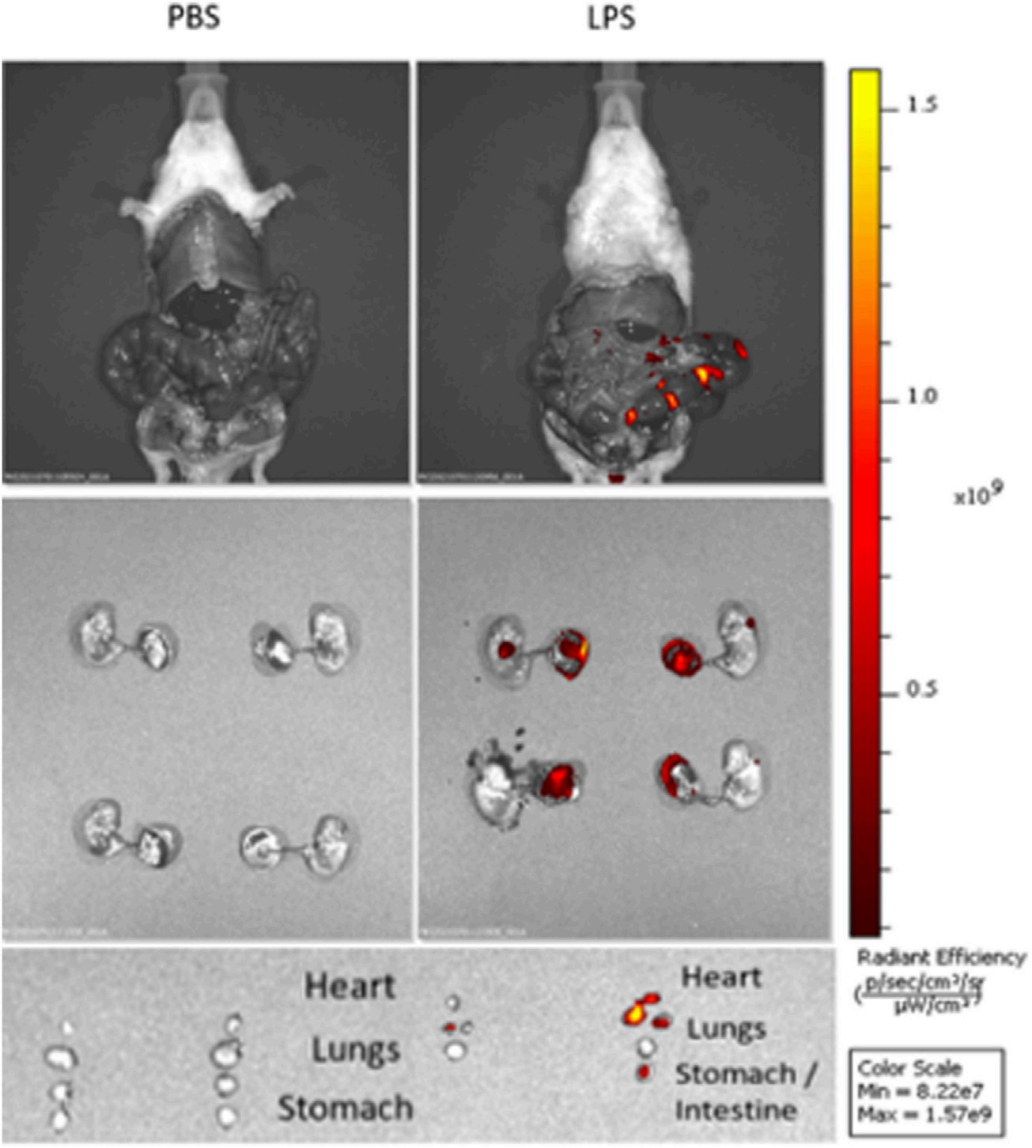
Intra-amniotic LPS localization and c-Myc expression in placenta. Distribution of LPS after intra-amniotic injections visualized by *in vivo* fluorescent imaging. Ultrasound-guided injections of LPS localized to amniotic cavity, fetal lungs and gastrointestinal tract.

To verify localization of LPS after US-guided IA injections we performed cesarean sections 3 h post-injection of Cy5.5-tagged LPS. *In vivo* and *ex vivo* imaging showed that LPS localized to the uterus without signal from the maternal abdominal cavity or circulation (Figure 2). After dissection, LPS was noted to be present on the fetal membranes and placenta as well as fetal lungs, heart, and gastrointestinal tract (Figure 2). These findings confirm the accuracy of US-guided injection in delivering LPS to the intra-amniotic cavity, simulating IAI.

### 3.2 IA LPS exposure modulates the transcriptome of placenta and fetal membranes

**FIGURE 3:**
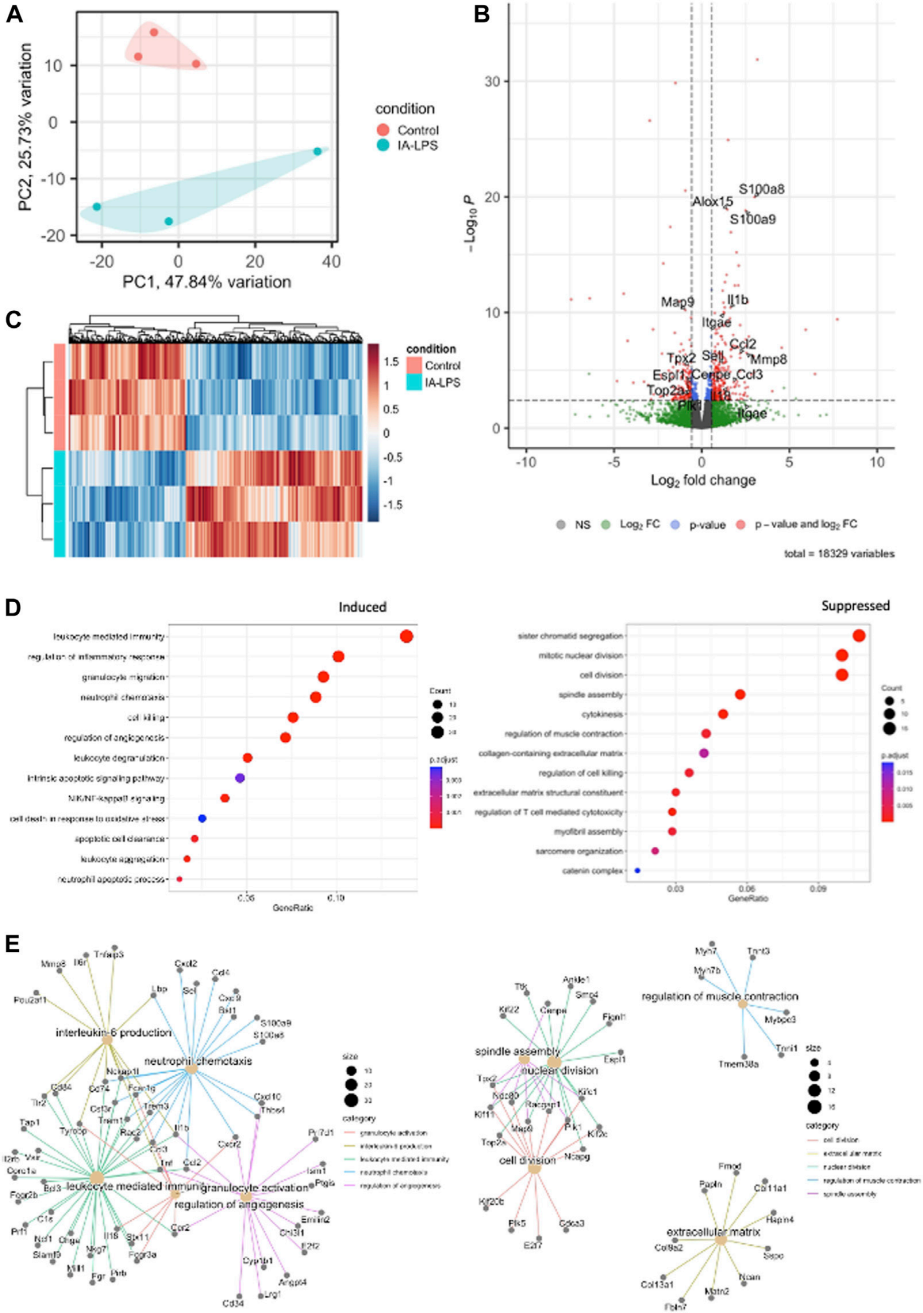
Intra-amniotic LPS changes the placental transcriptome. RNA-sequencing performed on placenta and fetal membranes sampled 24 h after LPS-exposure compared with controls showing: **(A)** Principal component analysis plot showing clear differentiation of gene expression between controls (pink) vs. LPS-exposed group (blue). **(B)** Heatmap of differentially expressed genes and **(C)** Volcano plot showing differential gene expression with genes of interest highlighted. Red–FDR<0.1 and fold change>1.5 **(D)** Dotplot showing related functions of genes that were differentially expressed between groups. LPS exposure induced genes associated with inflammation and leukocyte activation (*left*)*,* and suppressed genes associated with cell proliferation (*right*)*.* **(E)** Network plot of genes that were differentially induced (*left*) and suppressed (*right*) in LPS-exposed group compared to controls. *N* = 3 per group.

Bulk RNA-sequencing was performed on placenta and fetal membranes sampled 24 h post-LPS exposure and compared with controls of the same timepoint. We used PCA to identify global differences among samples on RNA-sequencing. PCA is a dimensionality reduction technique that allows quicker interpretation of the results while maintaining the maximum amount of information on each sample. There were large transcriptomic changes between groups with clear separation of LPS and control samples on PCA (Figure 3A). Differential expression analysis (Figures 3B, C, Supplementary Table S2) showed that IA LPS induced 271 and suppressed 165 genes relative to control. Overrepresentation analysis graphs show the *p*-value represented by the circle color, the number of differentially expressed genes belonging to each term represented by circle size, and the *x*-axis represents the gene ratio. Genes that were differentially expressed in LPS-exposed placenta and fetal membranes were associated with inflammation (*S100a8, S100a9, IL1b, Mmp8*), leukocyte activation (*Ccl2, Ccl3*), and decreased proliferation (*Map9, Tpx2*) (Figures 3C, D). Other differentially expressed genes were associated with regulation of angiogenesis, extracellular matrix organization and muscle contractility (Figure 3D). These findings confirm the induction of inflammation at the placenta in our model and suggest altered placental growth and development induced by IA LPS.

### 3.3 Prenatal c-Myc inhibition in IAI leads to increased c-Myc protein in the placenta and fetal membranes that are not neutrophil-driven

**FIGURE 4:**
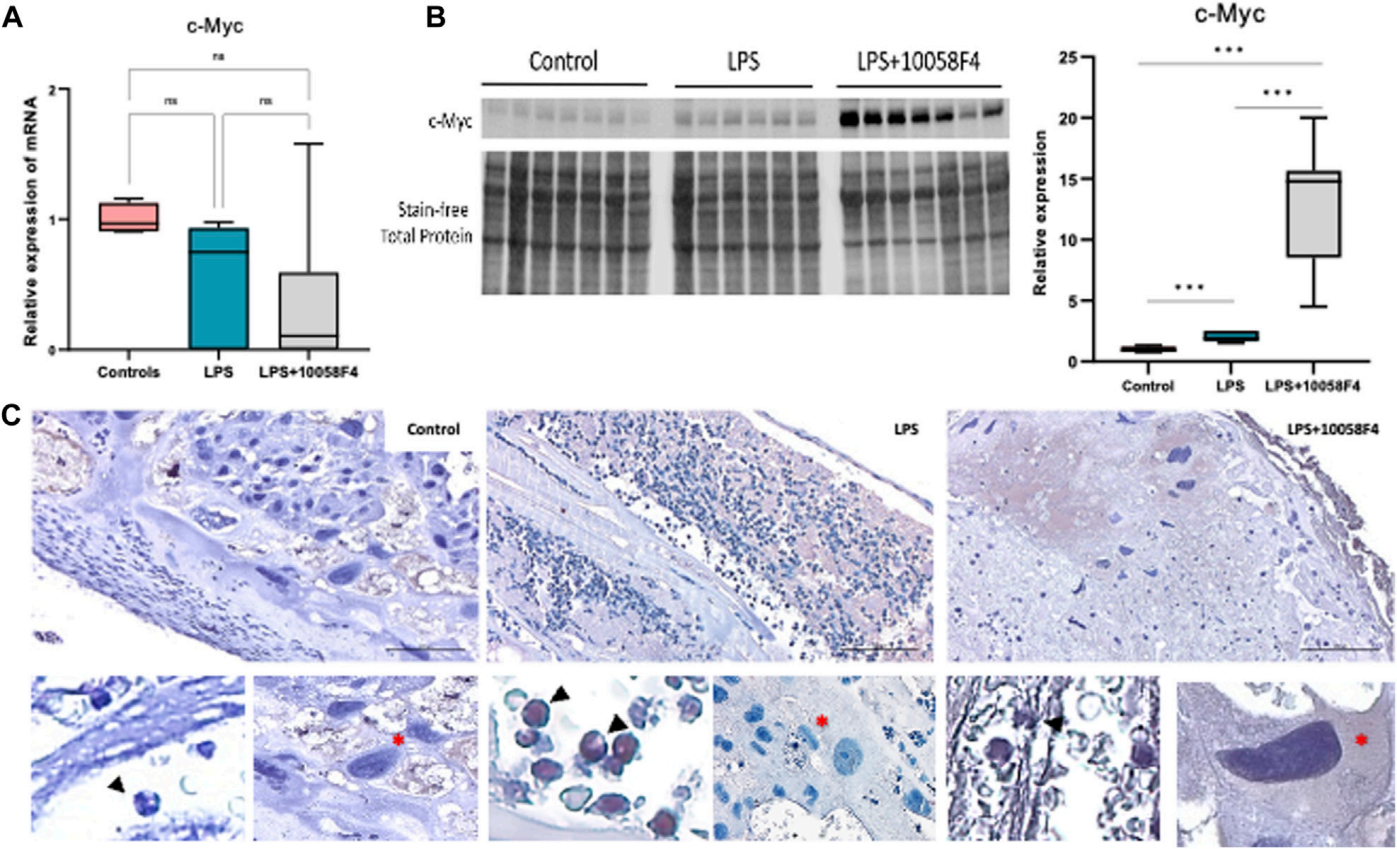
Prenatal c-Myc inhibition in IAI leads to increased c-Myc protein in the placenta and fetal membranes that are not neutrophil-driven. **(A)** RT-PCR performed on placenta and fetal membranes sampled at 24 h post-LPS exposure showed no significant changes. N = 4–8 per group. **(B)** Representative Western blot of placenta and fetal membrane lysates showing increased protein expression of c-Myc in LPS-exposed placenta and fetal membranes. With prenatal 10058-F4 c-Myc inhibitor treatment, c-Myc protein expression was further increased compared to both control and LPS groups. *N* = 6–8 per group *p < 0.05, ***p < 0.001, t-test, normalized to total protein. **(C)** Immunostaining of placenta sections with c-Myc antibody showing c-Myc expression (brown staining) in neutrophils (cropped and magnified, bottom left picture in each group, marked by black arrows) in LPS-exposed placenta. Prenatal c-Myc inhibition with 10058-F4 decreased neutrophil infiltration in LPS-exposed placenta and c-Myc was not expressed in neutrophils, but is expressed in cytoplasm of maternal decidual cells (cropped and magnified, bottom right picture in each group, marked by red asterisks).

To assess c-Myc expression in our model of IAI, we performed RT-PCR, Western blot, and immunostaining for c-Myc in the placenta and fetal membranes. There were no significant changes in mRNA expression of c-Myc in the placenta and fetal membranes sampled at 24 h post-LPS exposure in both IA LPS and IA LPS+10058-F4 treatment groups compared to controls (Figure 4A). The placenta and fetal membranes of IA LPS-exposed rats had increased c-Myc expression compared to controls by Western blot. Interestingly, prenatal c-Myc inhibitor treatment in IA LPS-exposed placenta and fetal membranes further induced c-Myc protein expression compared to both controls and IALPS groups (Figure 4B). On immunostaining, c-Myc was intranuclear and localized to neutrophils present at the maternal-fetal interface in the IA LPS-exposed group. In the LPS+10058F4 treatment group, c-Myc was not expressed in scant neutrophils that were present in the placenta and fetal membranes, but was expressed in the cytoplasm of placental decidual cells (Figure 4C). These findings show that c-Myc expression is induced by LPS and is expressed in the neutrophils in IAI. However, prenatal systemic 10058-F4 inhibitor treatment did not suppress c-Myc mRNA expression in the placenta and fetal membranes, but led to an increase in c-Myc protein expression in the placenta and fetal membranes which were not neutrophil-driven.

### 3.4 Prenatal systemic 10058-F4 treatment ameliorates inflammation and NET formation in placenta and fetal membranes induced by IA LPS

**FIGURE 5:**
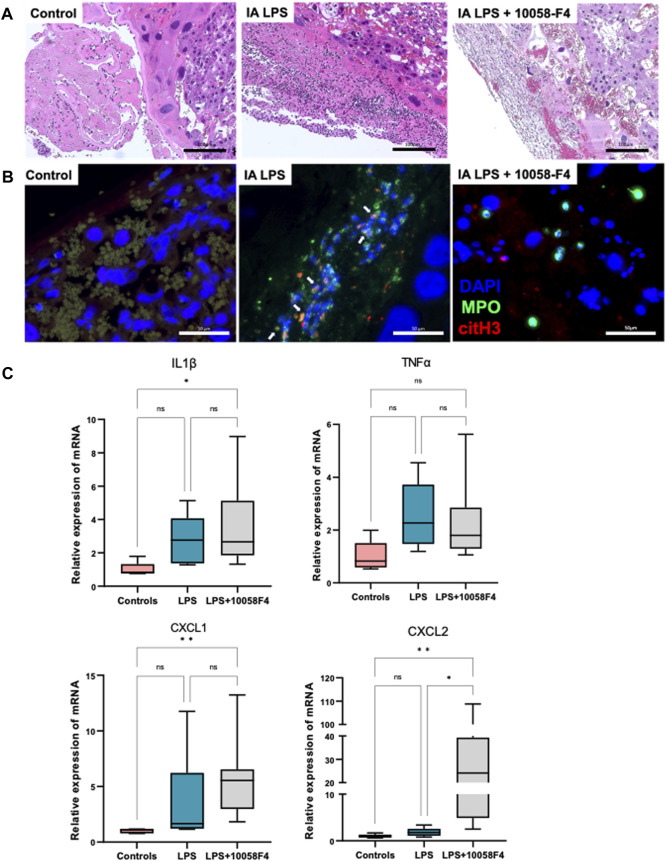
c-Myc inhibition decreases neutrophil infiltration, NET formation and modulates inflammation in placenta and fetal membranes. **(A)** Hematoxylin and eosin (H&E) staining of placenta sections showing increased neutrophil infiltration in placenta with LPS exposure when compared to controls, which was ameliorated with c-Myc inhibitor treatment. **(B)** Immunofluorescent staining of placenta sections with anti-myeloperoxidase (MPO) antibody (green) and anti-citrullinated histone (H3) antibody (red) showing increased NET formation in placenta with LPS exposure when compared to controls, which was ameliorated with c-Myc inhibitor treatment. **(C)** RT-PCR performed on placenta and fetal membranes sampled at 24 h post-LPS exposure. *N = 5*–*8 per group*. LPS exposure was not associated with significant changes in IL1β, TNFα, CXCL1 and CXCL2. Prenatal c-Myc inhibition in LPS-exposed pregnant rats induced IL1β and CXCL1 when compared to controls, and increased CXCL2 when compared to both control and LPS-exposed groups. *p < 0.05, **p < 0.01, Kruskal–Wallis test.

IA LPS induced neutrophil infiltration of the placenta and fetal membranes, confirming presence of histologic IAI (Figure 5A). IA LPS also induced NET formation assessed by immunofluorescence staining with colocalization of myeloperoxidase (MPO) and citrullinated histone-3 (citH3) (Figure 5B). Furthermore, LPS-exposed pregnant rats prenatally treated with 10058-F4 had decreased neutrophil infiltration and NET formation compared to pregnant rats exposed to IA LPS only (Figures 5A, B). Real-time PCR performed on placenta and fetal membranes sampled at 24 h after IA LPS injection showed no significant changes in mRNA expression of pro-inflammatory mediators IL1β, TNFα, CXCL1 and CXCL2 (Figure 5C). Prenatal c-Myc inhibitor treatment in the LPS-exposed group induced increases in mRNA expression of IL1β and CXCL1 compared to controls, and a very variable but overall significant increase in CXCL2 compared to both controls and the LPS-exposed group (Figure 5C). These findings show that c-Myc modulates inflammation of the placenta and fetal membranes.

### 3.5 Prenatal systemic 10058-F4 treatment decreases apoptosis and attenuates arrest of proliferation in the placenta induced by IA LPS

**FIGURE 6:**
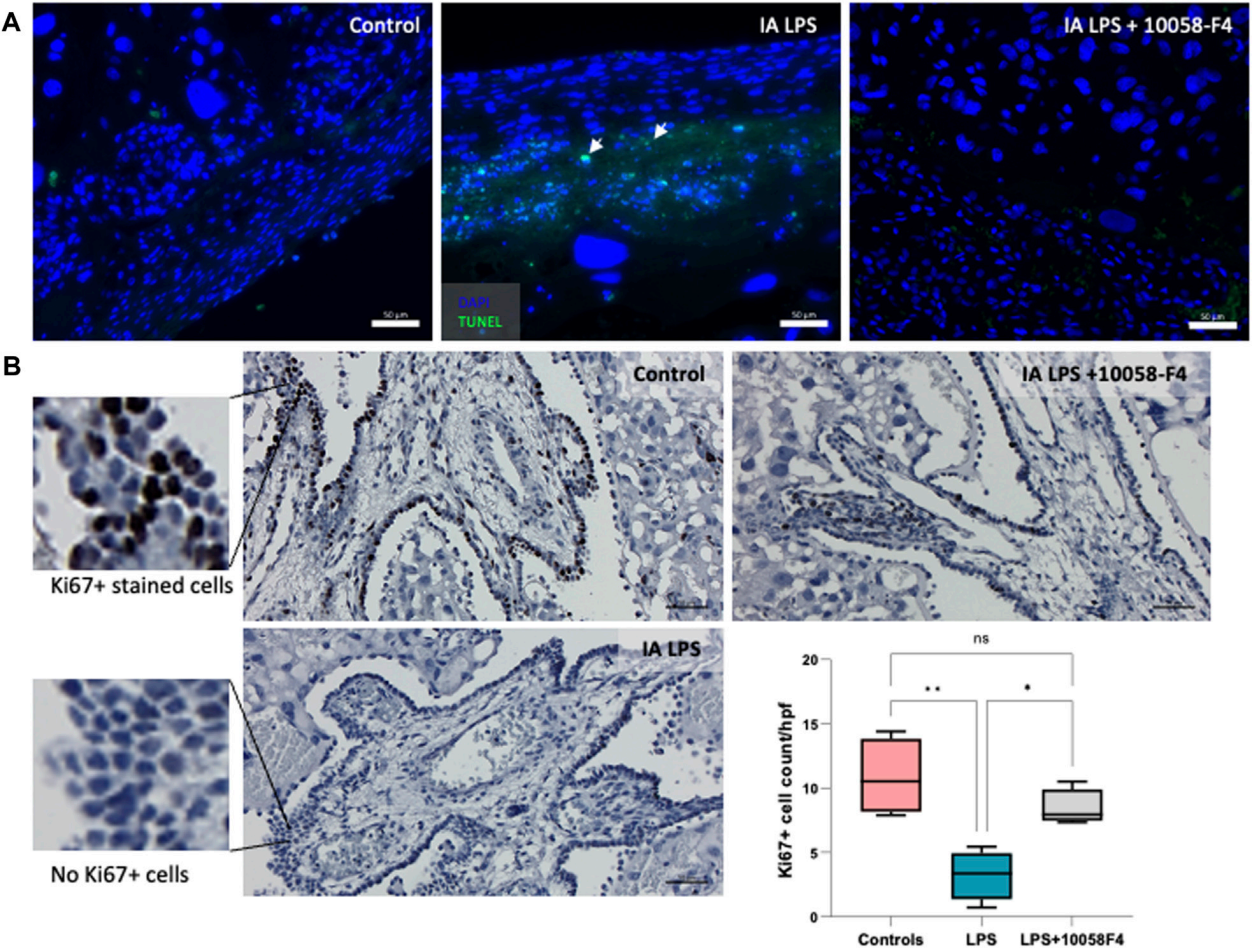
Prenatal systemic c-Myc inhibition decreases apoptosis and improves arrest of proliferation induced by LPS. **(A)** TUNEL assay performed on placenta sections showing increased apoptosis induced by LPS exposure when compared to controls, which was ameliorated with c-Myc inhibitor treatment. **(B)** Ki67 staining of representative placenta sections from each group showing decreased number of villous cytotrophoblasts positively stained with Ki67 (black stain) compared to controls, which improved with prenatal c-Myc inhibitor treatment. *p < 0.05, **p < 0.01, One-way Anova test. N = 4 per group.

IA LPS induced apoptosis in the placenta, assessed by TUNEL assay performed on placental sections (Figure 6A). When pregnant rats exposed to IA LPS were treated with c-Myc inhibitor 10058-F4, apoptosis was decreased compared to IA LPS only groups, similar to controls. Ki67 staining of placenta sections were used to assess cell proliferation, which were expressed in the villous cytotrophoblasts and most abundant in the control group (Figure 6B). Compared to controls, Ki67 staining was significantly decreased in placenta of pregnant rats that were exposed to IA LPS. However, when IA LPS-exposed pregnant rats were treated with 10058-F4, there was significantly increased number of Ki67+ cells per hpf compared to the IA LPS only group, and similar to controls.

### 3.6 IA LPS exposure modulates inflammation, collagen synthesis and extracellular matrix remodeling in the fetal lungs

**FIGURE 7:**
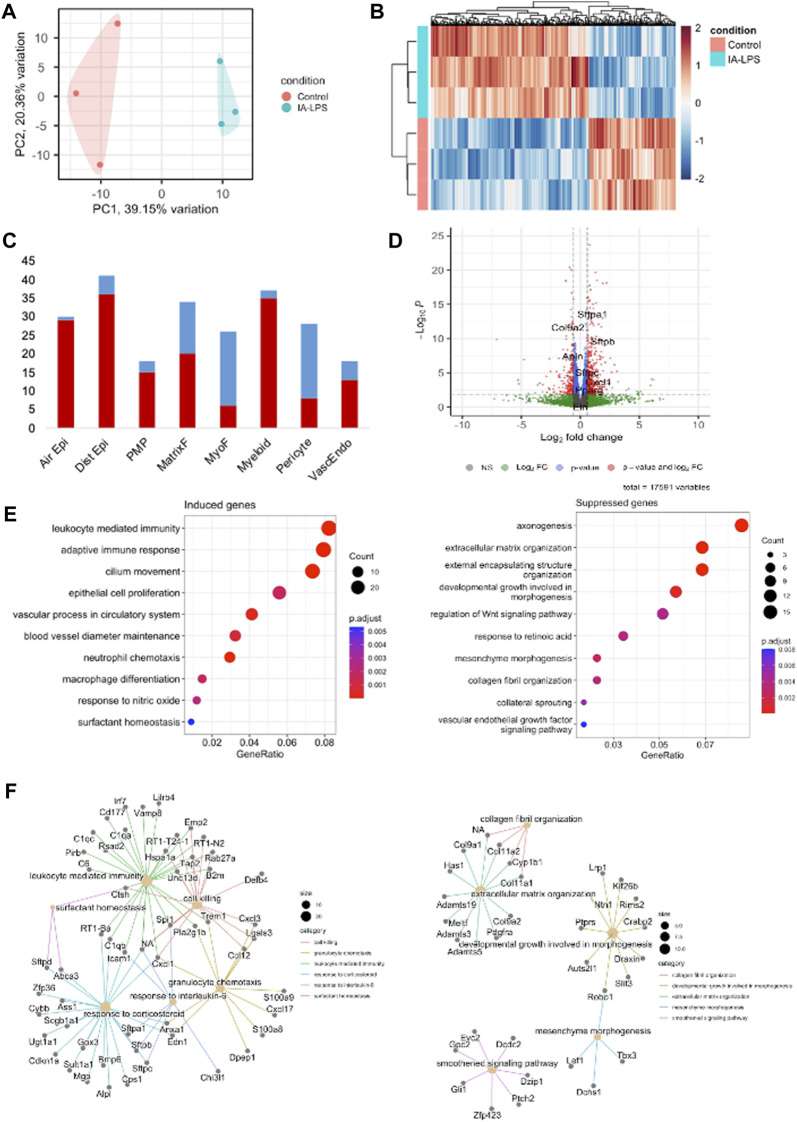
Intra-amniotic LPS induces fetal lung inflammation. Bulk RNA-sequencing of lungs at 24 h post-LPS exposure showing: **(A)** Principal component analysis showing clear differentiation of gene expression between groups. **(B)** Heatmap showing distinct differences in genes that were upregulated (*red*) and downregulated (*blue*) between control and LPS-exposed group. **(C)** Volcano plot showing differential gene expression with genes of interest highlighted. Red–significant. **(D)** Differential expression of genes by association with cell type. Red–induced. Blue–suppressed. *Air Epi–airway epithelium, Dist Epi–distal airway epithelium, PMP–proliferative mesenchymal progenitors, MatrixF–Matrix fibroflasts, VascEndo–Vascular endothelial.* **(E)** Chart showing related gene functions of genes that were differentially expressed between groups. Blue–Controls, orange–LPS-exposed. **(F)** Network plot of genes that were differentially induced (*left*) and suppressed (*right*) in LPS-exposed group compared to controls. N = 3 per group.

Bulk RNA-sequencing was performed on lung tissue obtained at 24 h. after IA LPS to characterize transcriptional changes induced by IAI on lung inflammation and remodeling. IA LPS induced large transcriptional changes to the lung with clear separation of groups by PCA (Figure 7A). There were significant differences in gene expression between controls and LPS-exposed groups with 379 genes induced and 209 genes suppressed by IA LPS (Figure 7B, Supplementary Table S3). We used data from single-cell RNA-seq of fetal lungs from LungMAP (Du et al., 2015; Ardini-Poleske et al., 2017) to determine cell-type specific signature genes. Differentially expressed genes in IA LPS exposed lungs were mapped to the signature gene list for the different cell populations identified by single-cell RNA-seq. IA LPS exposure induced signature genes for airway and distal epithelium cells, proliferative mesenchymal progenitors, and matrix fibroblasts, and suppressed signature genes for myofibroblasts, suggesting maturation of alveolar epithelial cells and suppression of myofibroblasts, which play a role in alveolar septation (Figure 7C). Gene set enrichment analysis of differentially expressed genes showed that genes suppressed by IA LPS were associated with collagen synthesis (*Col9a2, Col9a1, Col11a2, Col11a1*) and extracellular matrix organization (*Cxcl1*). On the other hand, genes induced by IA LPS were associated with chemokines that drive leukocyte migration to the fetal lung (*Cxcl3, Ccl12, Cxcl1, Cxcl13, Itgam, Lyz2, Mr1, Lgals3, Tap2, Trem1*), cell killing (*Cxcl3*, *Trem1*), and surfactant homeostasis (*Sftpa1*, *Sftpb*). Other notable genes that were differentially expressed were related to cell proliferation (*Cebpa*), angiogenesis (*Bmp6*) and inflammation (*Apln*, *Pparg*) (Figures 7D–F).

### 3.7 c-Myc is expressed in lung macrophages and is upregulated in normal postnatal lung development. IAI further increases c-Myc expression in postnatal lung development

**FIGURE 8:**
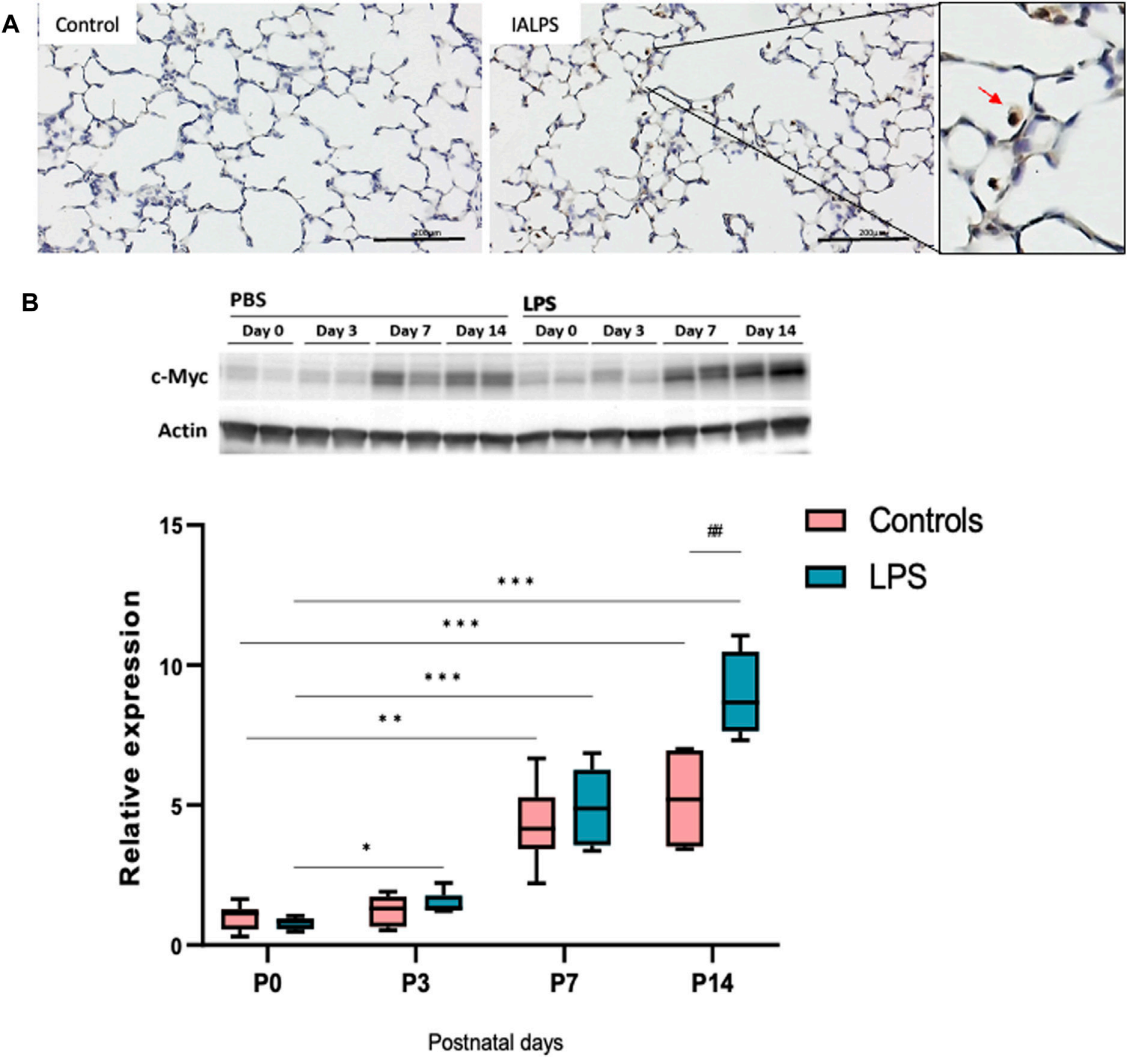
c-Myc is expressed in neonatal lung macrophages and is developmentally regulated. **(A)** Representative lung sections of rat pups at postnatal day 14 stained with c-Myc antibody showing localization of c-Myc expression to lung macrophages (brown-stained cells in magnified image on right). **(B)** Representative Western blot of lung tissue lysates showing expression of c-Myc over time (N = 4–7 per group). c-Myc was significantly upregulated during normal postnatal development, and this effect was more pronounced with exposure to LPS. *p < 0.02, **p < 0.002, ***p < 0.0002, ****p < 0.00002, ##*p* < 0.002.

Lung sections at P14 stained with c-Myc antibody showed c-Myc expression in lung macrophages of pups exposed to IA LPS (Figure 8A). c-Myc was upregulated in normal postnatal lung development, and LPS exposure further increased c-Myc expression in postnatal lung development. Western blot analysis of c-Myc expression in lungs of controls at various neonatal timepoints showed upregulation of c-Myc expression over time in the neonatal period, suggesting a role of c-Myc in postnatal lung development. IA LPS exposure exacerbated the normal upregulation in c-Myc expression compared to controls, suggesting that IA LPS modulates neonatal lung c-Myc expression (Figure 8B).

### 3.8 Fetal lung inflammation induced by IA LPS is transient and is modulated by c-Myc

**FIGURE 9:**
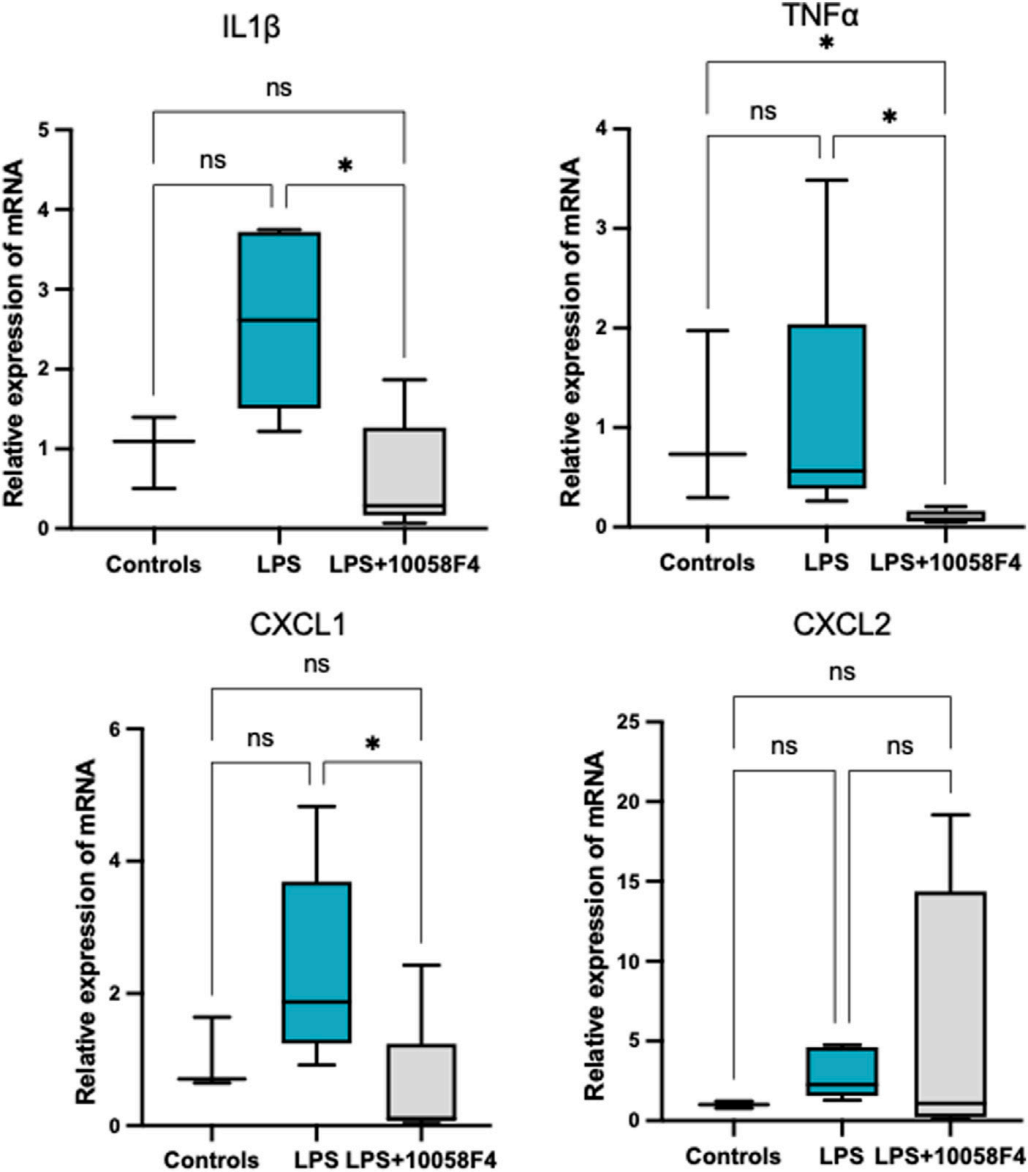
Fetal lung inflammation induced by IA LPS is transient and is modulated by c-Myc. RT-PCR of fetal lungs sampled at 24 h post-LPS exposure (N = 4–7 per group). There were no significant differences in mRNA expression of IL1β, TNFα., CXCL1 or CXCL2 with LPS exposure. Prenatal c-Myc inhibition in LPS-exposed fetal lungs suppressed IL1β and CXCL1 when compared to the LPS-exposed group and suppressed TNFα when compared to both LPS and LPS+10058F4 treated groups. There were no statistically significant changes in mRNA expression of CXCL2 with either LPS exposure alone or LPS+10058F54 treatment. *p < 0.05 Kruskal–Wallis test.

**FIGURE 10:**
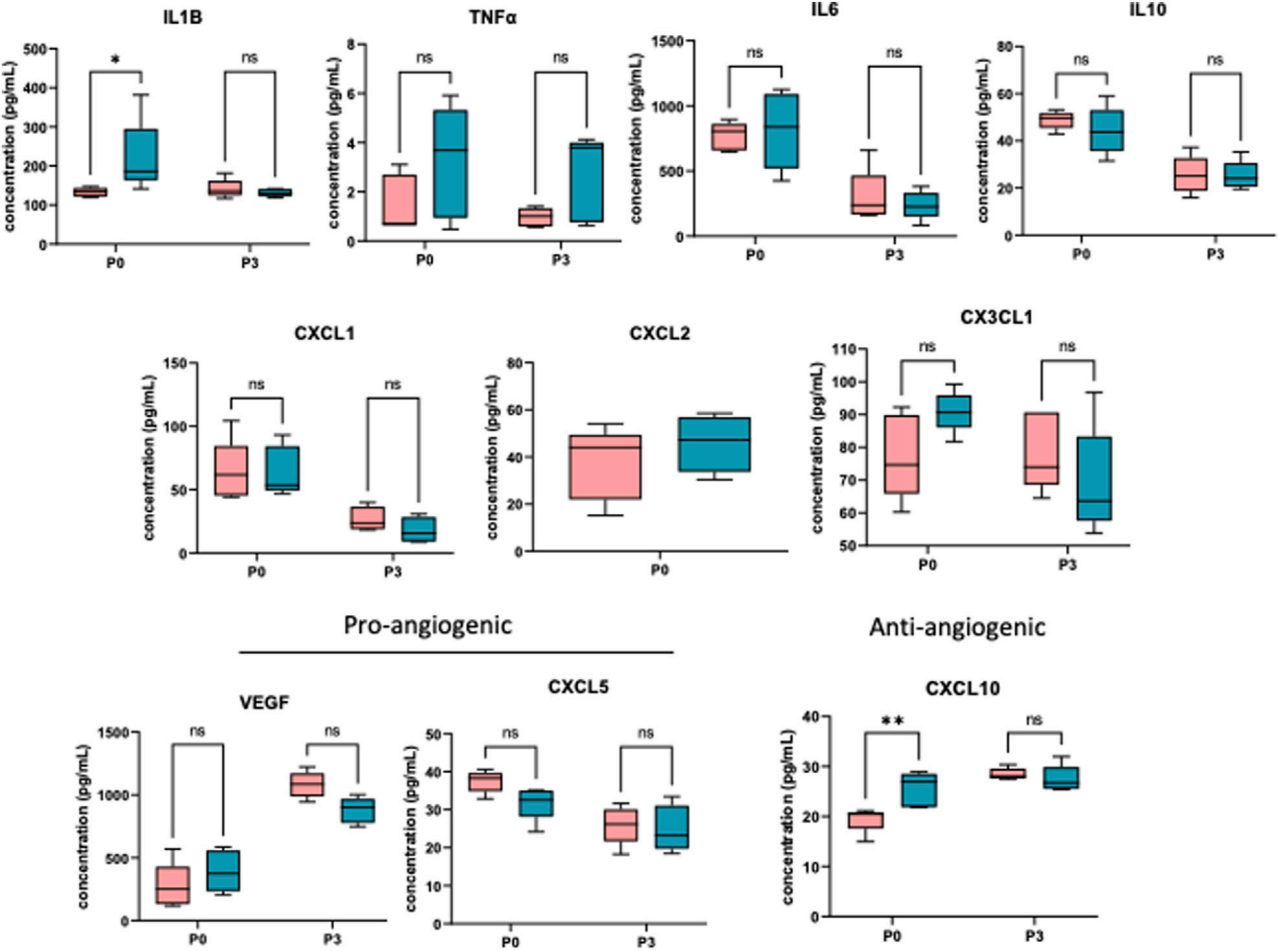
IA LPS induces fetal lung inflammation that resolves before postnatal day 3. Cytokine multiplex assay performed on neonatal lungs sampled on postnatal days (P) 0 and 3. In neonatal lungs at P0, LPS significantly induced IL1β and CXCL10. There were no statistically significant changes in TNFα, IL6, IL10, CXCL1, CXCL2, CX3CL1, CXCL5 and VEGF. N = 5 per group. *p < 0.05, **p < 0.02 Two-way Anova.

To determine the effect of prenatal c-Myc inhibition on fetal lung inflammation, we performed RT-PCR and cytokine multiplex assay. mRNA changes in IL1β, TNFα, CXCL1 and CXCL2 were not present at 24 h post-LPS exposure. Prenatal c-Myc inhibition in LPS-exposed fetal lungs suppressed IL1β and CXCL1 when compared to the LPS-exposed group, and suppressed TNFα when compared to both LPS and LPS+10058F4 treated groups. Similar to our findings in the placenta and fetal membranes, there was wide variability in mRNA expression of CXCL2 in response to LPS+10058F54 treatment, but these changes were not statistically significant when compared to control and LPS groups (Figure 9). On cytokine multiplex assay on lung lysates, LPS induced increased IL1β and CXCL10 at P0 only, and there were no significant changes in other pro-inflammatory mediators TNFꭤ, IL6, IL10, CXCL1, CXCL2, CX3CL1 or CXCL5 on P0 or P3 (Figure 10). There were no significant changes in angiogenic factor VEGF induced by LPS. These findings demonstrate that fetal lung inflammation induced by IA LPS is transient and resolves before postnatal day 3.

### 3.9 Prenatal c-Myc inhibition improves LPS-induced changes associated with BPD

**FIGURE 11:**
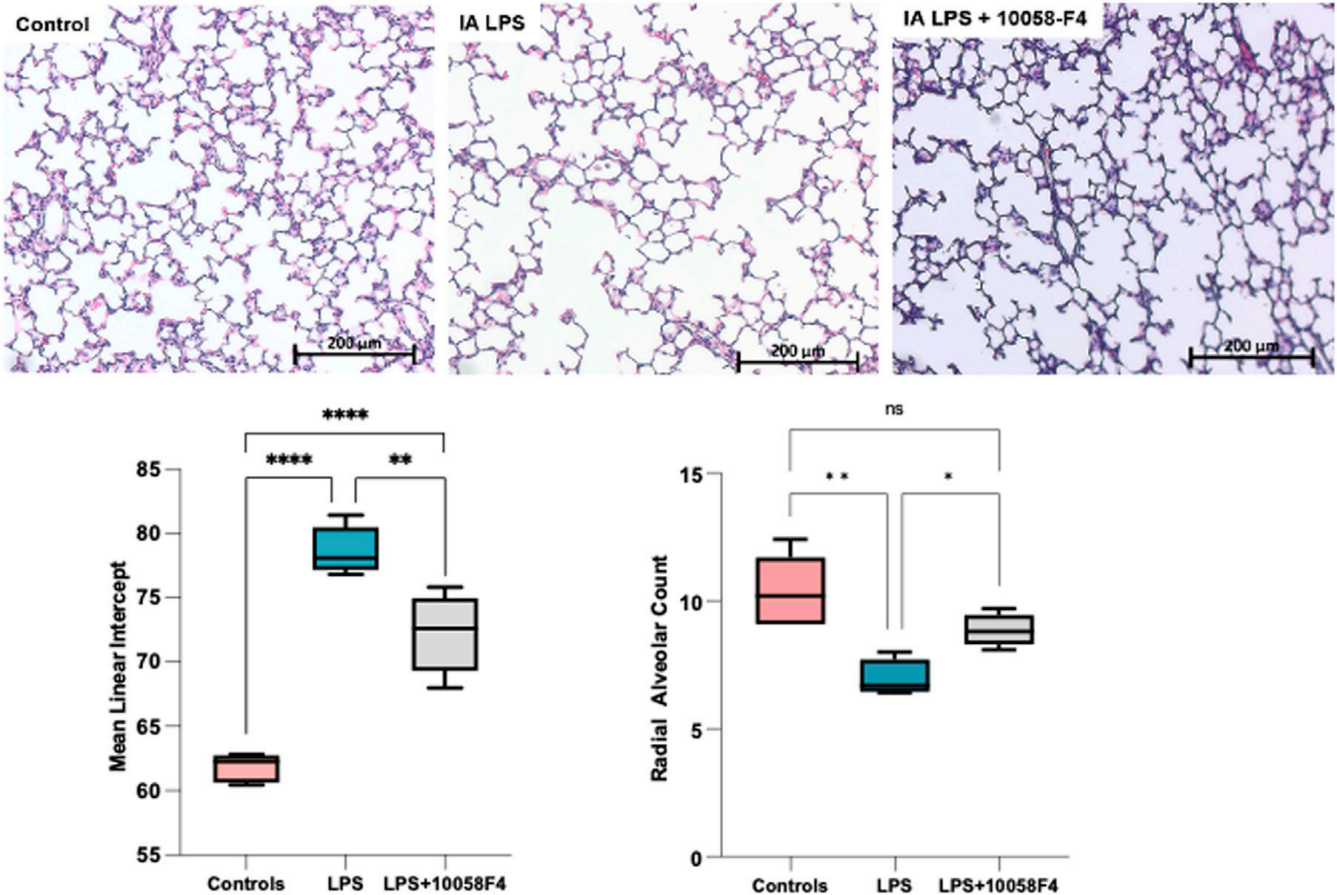
c-Myc inhibition improves alveolarization and pulmonary hypoplasia induced by IAI. Representative lung sections of rat pups at postnatal day 14 (n = 5 per group) stained with H&E to assess alveolarization. Mean linear intercept was significantly increased and radial alveolar counts were significantly decreased in IALPS-exposed lungs compared to controls at postnatal day 14, suggesting alveolar simplification and pulmonary hypoplasia. These effects were significantly decreased with c-Myc inhibitor treatment. *p < 0.05, **p < 0.005, ****p < 0.0001.

**FIGURE 12:**
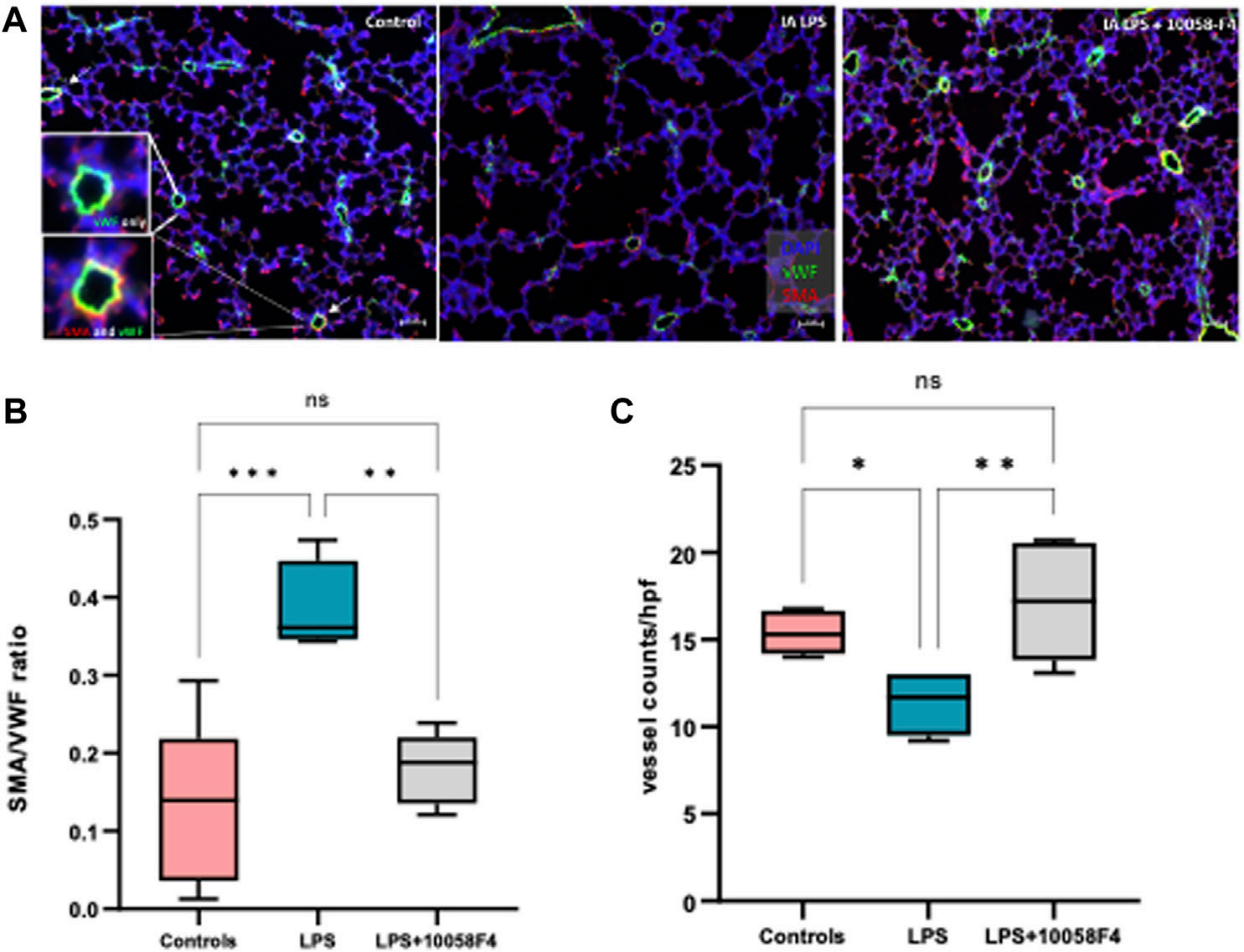
c-Myc inhibition increases angiogenesis and decreases pulmonary vascular remodeling in IAI at P14. **(A)** Immunostaining of lung sections sampled at postnatal day 14 with von Willebrand factor (vWF–green) and smooth muscle actin (SMA–red) (n = 4-8 per group) **(B)** Quantitative analysis of pulmonary vascular muscularization by calculating ratio of vessels stained with both SMA and vWF (demonstrated by white arrows and magnified images of vessels on far left), to number of vessels stained with vWF only (SMA/vWF ratio), and **(C)** quantitative analysis of angiogenesis using number of vessels stained with vWF per hpf. LPS exposure decreased angiogenesis and increased pulmonary vascular muscularization. Prenatal c-Myc inhibition with 10058-F4 treatment improved angiogenesis and decreased pulmonary vascular muscularization induced by LPS.

To assess alveolarization, lung sections obtained at P14 were stained with H&E and analyzed for the mean linear intercept (MLI) and radial alveolar count (RAC), which are measures of alveolar septation and quantification of lung airspaces. MLI was increased in LPS-exposed neonatal rats at P14, demonstrating alveolar simplification relative to controls. RAC was significantly decreased in LPS-exposed neonatal rats (Figure 11). To assess pulmonary vascular muscularization, lung sections were double-stained with von Willebrand factor (vWF) and smooth muscle actin (SMA) (Figure 12A), and the ratio of number of vessels stained with SMA-vWF to the number of vessels stained with vWF was calculated (Figures 12B, C). In IA LPS-exposed lungs at P14, there was an increased ratio of SMA/vWF-stained vessels, indicating increased pulmonary vascular remodeling, which occurs in pulmonary hypertension. Compared to pregnant rats exposed to LPS only, prenatal treatment of LPS-exposed pregnant rats with c-Myc inhibitor 10058-F4 led to improved alveolarization, increased angiogenesis and decreased pulmonary vascular muscularization induced by LPS (Figures 11, 12). These findings suggest that c-Myc has a role in modulating impaired lung development induced by IAI.

## 4 Discussion

To understand the role of c-Myc on placental inflammation and BPD induced by IAI we used ultrasound-guided IA LPS injections and allowed natural delivery, eliminating confounding effects of stress and inflammation induced by maternal laparotomy. This rodent model of IAI has been validated to cause a subclinical syndrome of IAI, which is most clinically relevant, compared to intra-uterine or intraperitoneal injections (Gomez-Lopez et al., 2018). We further validated this model with demonstrated uptake of tagged LPS in the amnion, fetal lungs, and gut, confirming exposure of the fetus to intraamniotic inflammation through the fetal membranes, lungs, and gastrointestinal tract. These findings are consistent with large animal models of IAI using IA LPS (Kramer et al., 2010). Overall, our results suggest that c-Myc drives neutrophil infiltration in IAI, and that c-Myc has a role in modulating neonatal lung alveolar development and pulmonary vascular remodeling.

Bulk RNA-sequencing of placenta and fetal membranes showed transcriptional changes in genes associated with inflammation (*S100a8, S100a9, Alox15, Il1b, Mmp8*), leukocyte activation (*Ccl2, Ccl3*), and decreased proliferation (*Map9, Tpx2*) in response to LPS exposure. *S100a8*, *S100a9, Il1b,* and *Mmp8* have been strongly associated with fetal inflammatory response syndrome (FIRS), preterm labor, and chorioamnionitis in preterm infants (Kallapur et al., 2013; Holmstrom et al., 2019; Golubinskaya et al., 2020), further supporting our rat model using IA LPS injections to simulate IAI and a FIRS-like response. *Alox15* encodes for arachidonic acid through the lipoxygenase 15 (ALOX15) pathway, which participates in glucocorticoid receptor response and modulates parturition through prostaglandin E2 synthesis pathway, suggesting a potential role in inflammation induced preterm labor (Zhang et al., 2023). *Ccl2* and *Ccl3* code for known inflammatory chemokines that are elevated in chorioamnionitis-exposed preterm infants (Stepanovich et al., 2023). LPS exposure suppressed *Map9* (Microtubule-Associated Protein 9) and *Tpx2* (Targeting protein Xklp2), which are protein-coding genes that are involved in cell growth and division. *Tpx2* abnormalities result in abnormal spindles and meiosis, and chromosome segregation errors in animal studies, which may be associated with birth defects and pregnancy loss (He et al., 2022; Zhang et al., 2022). These transcriptomic changes suggest a negative impact of placental growth and development induced by IA LPS.

LPS induces inflammation in the placenta and fetal membranes within 24 h of exposure, demonstrated by increased neutrophil infiltration and NET formation. Neutrophil infiltration is a hallmark of IAI, and neutrophil recruitment with NET formation are immune defense mechanisms against infections or danger signals (Gomez-Lopez et al., 2017). However, excessive neutrophil infiltration and NET formation in pathologic conditions may exacerbate tissue injury (Sorensen and Borregaard, 2016). In LPS-exposed placenta and fetal membranes, c-Myc is expressed in the nuclei of neutrophils. Prenatal systemic c-Myc inhibition with 10058F4 decreased LPS-induced neutrophil infiltration and NET formation in the placenta and fetal membranes, suggesting that c-Myc regulates inflammation in the placenta and fetal membranes in our rat model of IAI, and that c-Myc modulates NET formation in the placenta. Interestingly, c-Myc was not expressed in the neutrophils in LPS-exposed placenta treated with 10058F4 but is expressed in the cytoplasm of maternal decidual cells. The presence of c-Myc in the placenta has only been described in limited studies, in human choriocarcinoma and hydatidiform moles (Diebold et al., 1991; Cheung et al., 1993; Fulop et al., 1998). There is limited data on the role of c-Myc in the placenta in normal pregnancies and in IAI. On the other hand, the differences between intranuclear expression of c-Myc *versus* cytoplasmic c-Myc expression have been described, and the biological functions of c-Myc differ when expressed in nuclei or cytoplasm (Conacci-Sorrell et al., 2010). The expression of c-Myc in nuclei or cytoplasm has been used to risk stratify and prognosticate cancers (Geisler et al., 2004; Conacci-Sorrell et al., 2010; Gong et al., 2017).

We administered the c-Myc inhibitor 10058-F4 systemically, but it is not known whether 10058-F4 crosses the placenta. Since we performed ultrasound-guided intra-amniotic LPS injections to minimize systemic effects and to mimic subclinical chorioamnionitis, inflammation is localized to the amniotic sacs, placenta, and fetus. LPS is a toll-like receptor 4 (TLR4) agonist, and has been shown to prevent degradation of c-Myc via activation of the TLR/MyD88 pathway, but the exact mechanism by which LPS induces neutrophil-targeted c-Myc in the placenta are unknown (Wang et al., 2014). Our findings suggest that prenatal systemic 10058F4 treatment leads to inhibition of c-Myc in the maternal circulation, attenuating recruitment of neutrophils to the placenta and fetal membranes in IAI. However, prenatal 10058F4 did not inhibit c-Myc expression in the maternal decidual cells, which is likely constitutional in the placenta and fetal membranes and likely related to other functions regulated by c-Myc, such as cellular proliferation. The overall increase in c-Myc expression in the placenta and fetal membranes treated with 10058-F4 is not neutrophil-driven and may be a compensatory mechanism to overcome prenatal c-Myc suppression in cell types other than neutrophils, and possibly to protect the pregnancy and fetus. The role of c-Myc in placental and fetal development, and in IAI needs to be further explored.

We did not observe statistically significant changes in mRNA expression of specific inflammatory cytokines and chemokines (IL1β, TNF-α, CXCL1 and CXCL2) induced by LPS in the placenta and fetal membranes. This is likely due to lack of statistical power as there was an uptrend in IL1β and TNFα which corroborates with RNA-sequencing data. However, prenatal c-Myc inhibition in the LPS-exposed group significantly induced IL1β, CXCL1 and CXCL2 when compared to controls. CXCL1 and CXCL2 are members of the CXC chemokine subfamily that participate in wound healing, immunoregulation and neutrophil recruitment through activation of a CXCR2 receptor (Sawant et al., 2021). CXCR2 antagonism has been shown to decrease c-Myc expression in bone marrow of patients with chronic myeloid leukemia, through a CXCR2-mTOR-c-Myc cascade (Kim et al., 2021). c-Myc also regulates programmed cell death-ligand 1 (PD-L1), and c-Myc inhibition with 10058F4 in esophageal cancer cells downregulated PD-L1 (Liang et al., 2020). PD-L1 deficiency in neutrophils has been shown to lead to impaired secretion of CXCL1 and CXCL2 (Yu et al., 2022). In pregnancy, CXCL1 is produced in the placenta and participates in implantation, placentation and decidual angiogenesis (Korbecki et al., 2022). The dynamics between CXCL1, CXCL2 and CXCR2 receptor activation have been shown to be complex, and together, are vital in achieving homeostasis of inflammation and tissue healing (Sawant et al., 2021). CXCL1 and CXCL2 elevation in pregnant mice have been shown to be associated with massive decidual neutrophil infiltration and fetal loss (Mizugishi et al., 2015). The mechanisms by which c-Myc directly modulates CXCL1 and CXCL2, and their roles in IAI are unknown and need to be further explored. In our experiments, we observed that LPS exposure with prenatal c-Myc inhibition in a pregnant model induces an imbalance in CXCL1 and CXCL2 expression in the placenta and fetal membranes. Regardless, we observed reduction of neutrophil infiltration and NET formation with prenatal c-Myc inhibition, associated with improved neonatal lung remodeling. More studies are required to investigate the relationship between c-Myc, chemokine balance in pregnancy and effects on fetal development.

In fetal lungs, bulk RNA-sequencing showed that LPS exposure induced genes associated with chemokines that drive leukocyte migration to the fetal lung (*Cx3cr1, Cxcl3, Ccl12, Cxcl1, Cxcl13, Itgam, Lyz2, Mr1, Lgals3, Tap2, Trem1*), inflammation (*Angptl4, Chi3l1*), and surfactant homeostasis (*Sftpa1*, *Sftpb, Ctsh*). *Cx3cr1* encodes for the receptor of fractalkine/CX3CL1, which is a chemokine involved in adhesion and migration of leukocytes. CX3CL1-CX3CR1 axis is strongly associated with inflammatory lung diseases (Zhang and Patel, 2010). CX3CR1 is also a major receptor for respiratory syncytial virus infections and has been found to modulate airway inflammation and mucus production (Das et al., 2017), as well as LPS-induced lung injury through NFκB activation (Ding et al., 2016). *Cxcl1, Cxcl3, Cxcl13, Itgam, Lyz2, Lgals3* encode for cytokines that are known to be dysregulated in animal models of hyperoxia-induced BPD (Deng et al., 2000; Rudloff et al., 2017; Hurskainen et al., 2021; Dong et al., 2022)*,* and *Trem1* is a protein-coding gene encoding for Triggering Receptor Expressed on Myeloid Cells 1 (TREM1) which is upregulated in preterm infants who developed BPD (Ambalavanan et al., 2009). *Angptl4* has been shown to be dysregulated in inflammation and may have protective anti-inflammatory and anti-angiogenic effects through modulation of NF-kBp65 and IL6 expression (Wang et al., 2013). *Angptl4* gene knockout in mice models of LPS-induced lung injury decreased inflammation and tissue damage, and improved recovery and mortality, suggesting a significant role of *Angptl4* in the mechanisms of inflammation-induced lung injury (Guo et al., 2015; Li et al., 2015). *Chi3l1* has been implicated in pulmonary fibrosis and is expressed in lung alveolar macrophages and regulates inflammation, cell proliferation and apoptosis in connective tissue cells including fibroblasts (Recklies et al., 2002). *Ctsh* is involved in pulmonary surfactant protein B production and plays a vital role in lung development (Lu et al., 2007; Buhling et al., 2011). Similar to our findings in the placenta, RT-PCR of IL1β, TNF-α, CXCL1 and CXCL2 in the fetal lungs did not show statistically significant differences induced by LPS. However, in fetal lungs exposed to LPS+10058F4, we observed suppression in IL1β and CXCL1 compared to LPS-exposed group, and suppression in TNF-α compared to both controls and LPS-exposed group. IL1β, TNF-α and CXCL1 dysregulation are well-associated with IAI and BPD (Ambalavanan et al., 2009; Cappelletti et al., 2020; Heydarian et al., 2022).

Cytokine multiplex of neonatal lungs showed that IA LPS significantly induced IL1β and CXCL10 at P0. There were no statistically significant changes in TNFα, IL6, IL10, CXCL1, CXCL2, CX3CL1, CXCL5 and VEGF. IL1β is a well-known major modulator in IAI and bronchopulmonary dysplasia (Bry et al., 2007; Cappelletti et al., 2020). CXCL10 participates in inflammation, specifically macrophage infiltration, and modulates migration of vascular smooth muscular cells and endothelial cell permeability (Li et al., 2021). It is upregulated in tracheal aspirates of preterm infants with BPD and is strongly associated with idiopathic pulmonary arterial hypertension in adults (Aghai et al., 2013; Li et al., 2021; Cunningham et al., 2022). Inhibition of CXCL10 in animal models have been shown to improve pulmonary hypertension and LPS-induced lung injury (Lang et al., 2017; Cunningham et al., 2022). Collectively, our RNA-sequencing data showed that IA LPS induced inflammatory transcriptional changes in the fetal lungs which persisted through postnatal day 0, but these inflammatory changes appeared to be transient and resolved before postnatal day 3. However, prenatal systemic c-Myc inhibition suppressed mRNA expression of inflammatory cytokines in the fetal lungs.

At P14, rat pups exposed to LPS had alveolar simplification and pulmonary hypoplasia, decreased angiogenesis, and increased pulmonary vascular muscularization, consistent with a BPD-phenotype. Extracellular matrix (ECM) remodeling is another important component in the pathophysiology of BPD, and RNA sequencing of fetal lungs at 24 h after LPS exposure showed significant modulation of genes related to ECM remodeling (*Cxcl1*) and organization, collagen synthesis (*Col9a2, Col9a1, Col11a2, Col11a1*), proliferative mesenchymal progenitors and matrix fibroblasts. These results are consistent with previously reported data where extracellular matrix development and collagen protein synthesis was disrupted in lungs of preterm rhesus macaques exposed to IALPS (Schmidt et al., 2020). Targeted inhibition of extracellular matrix proteins provides partial protection from lung injury induced by hyperoxia and anti-inflammatory treatment in animal models improved changes related to BPD through modulation of collagen and extracellular matrix protein expression and TGF-β1/Smads pathway, suggesting potential for targeted therapy in modifying abnormal extracellular matrix remodeling in IAI-induced BPD (Mizikova et al., 2018; Chen et al., 2020).

c-Myc expression in rat fetal lungs have been shown to be elevated during a period of growth, then decrease over increasing gestational age, coinciding with the time of cell differentiation and beginning of surfactant production (Kellogg et al., 1992). Interestingly, protein expression of c-Myc increases postnatally in neonatal lungs of control pups, demonstrating a role of c-Myc in normal postnatal lung development. c-Myc is an important regulator of cell proliferation, differentiation, and growth. When exposed to LPS, c-Myc is overexpressed during neonatal lung development, which is also associated with large transcriptomic differences in cell differentiation and proliferation, airway and alveolar epithelial cell differentiation, collagen and extracellular matrix organization, and surfactant proteins, predicting abnormal lung development, which is consistent with the lung parenchymal and vascular changes observed in our 14-day old pups. In LPS-exposed rat pups, prenatal c-Myc inhibition improved alveolarization, increased angiogenesis and decreased pulmonary vascular muscularization, demonstrating a role of c-Myc in IAI-associated BPD. The exact mechanisms by which c-Myc induces neonatal lung remodeling need to be further explored.

BPD with pulmonary hypertension is highly clinically relevant. A subset of preterm infants with moderate to severe BPD develop pulmonary hypertension (BPD-PH), which is associated with significantly increased morbidity and mortality (Hansmann et al., 2021). Compared to infants without pulmonary hypertension, infants with BPD-PH have higher rates of tracheostomy, need for tube feeds, poorer growth and neurodevelopmental outcomes, and hospital readmissions (Nakanishi et al., 2016; Al-Ghanem et al., 2017; Lagatta et al., 2018). Pulmonary hypertension is independently associated with exposure to IAI (Woldesenbet and Perlman, 2005; Yum et al., 2018). In cord blood of newborns exposed to IAI, there is an imbalance of angiogenic factors such as sFlt-1, VEGF, and endothelial progenitor cells. In preterm rhesus macaque fetuses, exposure to intra-amniotic LPS results in large transcriptional changes of genes regulating vascular development (Schmidt et al., 2020). In preterm lamb models, IA LPS exposure causes decreased pulmonary blood flow and increased pulmonary arterial pressures, suggesting a direct link between IAI and pulmonary hypertension (Polglase et al., 2010). The mechanisms by which pulmonary hypertension is associated with BPD and IAI are not completely understood, and given the significant burden of BPD-PH, there is a pressing need to investigate the mechanistic pathways and potential interventions to improve neonatal outcomes. Our results suggest that c-Myc regulate neonatal lung vascular remodeling in response to IAI.

We recognize limitations in our study. There is variance in RNA sequencing data within the control animals, which may be attributed to the fact that sterile PBS was injected in controls as a placebo under clean, but not sterile, conditions. Thus, the procedure by itself may induce an intra-uterine inflammatory response which may account for the data variance. Also, we do not have RNA-sequencing available for the LPS-exposed group treated with c-Myc inhibitor 10058-F4. Given the various perinatal interventions and treatment, there was rejection and death of pups in LPS-exposed and treated groups which could influence the results. Further animals treated with 10058-F4 were done in different days from control and IA LPS only animals, which could have introduced batch effect in our analysis. All animals were purchased from the same vendor and the same lot of LPS was used in all experiments.

Despite these limitations, we can conclude that the transcription factor c-Myc is dysregulated in the placenta, fetal membranes, and neonatal lungs in intra-amniotic inflammation and modulates inflammation of the placenta and fetal membranes in the rat model of IAI induced by IA LPS and modulates IAI-induced neonatal lung remodeling. Further studies are needed to explore the mechanisms by which c-Myc modulates NET formation, and to investigate c-Myc as a potential therapeutic target in IAI, and IAI-induced BPD. However, c-Myc has been challenging to target therapeutically due to its intranuclear nature and its highly disordered structure (Llombart and Mansour, 2022). Moreover, it is a ubiquitous transcription factor that is involved in multiple cell processes vital to physiologic functions. The ideal c-Myc inhibitor needs to be highly selective to diseased conditions and normalize c-Myc levels, rather than fully inhibiting all c-Myc-associated functions. Novel c-Myc inhibitors with such properties have since become available and should be the focus of future studies to investigate translational therapeutic potential of c-Myc inhibition to improve adverse neonatal outcomes induced by antenatal inflammation.

## Data Availability

The gene expression data have been deposited in NCBI’s Gene Expression Omnibus (GEO: https://ncbi.nlm.gov/geo/) and are accessible through GEO Series accession numbers GSE237595 and GSE239349.
