# Supplementary material for: c-Myc Drives inflammation of the maternal-fetal interface, and neonatal lung remodeling induced by intra-amniotic inflammation
Source: Front Cell Dev Biol. 2024 Feb 28;11:1245747. doi: 10.3389/fcell.2023.1245747 (PMC10933046; doi:10.3389/fcell.2023.1245747)
Supplement: Supplementary file 3 [file Table1.DOCX]

| A. Primary Antibodies and Dilutions |
| --- |
| Myc Antibody (9E10): sc-40 (Santa Cruz Biotechnology) WB 1:500, IHC 1:100 |
| Beta-actin Mouse (Sigma-Aldrich) #A5441 WB 1:10000 |
| Anti-Histone H3 (citrulline R2 + R8 + R17) antibody Catalog #ab5103 (Abcam) IF 1:50 |
| Human/Mouse Myeloperoxidase/MPO antibody Catalog # AF3667 (R&D Systems) IF 1:50 |
| von Willebrand factor, #A0082 (Dako) IF 1:50 |
| Monoclonal Anti-Actin, α-Smooth Muscle, #A2547 (Sigma-Aldrich) IF 1:100 |
| B. TaqMan® Gene Expression Assays (ThermoFisher Scientific) |
| TNF-alpha Catalog # 4331182 Assay ID Rn01525859_g1 |
| IL-1b Catalog # 4331182 Assay ID Rn99999009_m1 |
| Cxcl2 Catalog # 4331182 Assay ID Rn00586403_m1 |
| Cxcl1 Catalog # 4331182 Assay ID Rn00578225_m1 |
| c-Myc Catalog # 4331182 Assay ID Rn07310910_m1 |
| Eukaryotic 18S rRNA Endogenous Control (FAM™/MGB probe, non-primer limited) Catalog # 4333760F |
